# Development, Factor Structure, and Psychometric Validation of the Impostor Phenomenon Assessment: A Novel Assessment of Impostor Phenomenon

**DOI:** 10.1177/10731911221141870

**Published:** 2023-01-02

**Authors:** Deanna L. Walker, Donald H. Saklofske

**Affiliations:** 1 University of Western Ontario, London, Ontario, Canada

**Keywords:** impostor phenomenon, imposter syndrome, assessment, personality, psychometric validation

## Abstract

Despite growing attention surrounding impostor phenomenon (also known as “imposter syndrome”), recent reviews have suggested that current measures may be inadequate in capturing the complex and multifaceted nature of this construct. The objective of the current studies was to clarify the theoretical conceptualization of impostor phenomenon based on experiences in an achievement-oriented setting. We conducted a review of the literature and developed an item pool for a novel impostor phenomenon assessment (IPA) (Study 1). Exploratory factor analyses (Study 1) and confirmatory factor analyses (Study 2) assessed this initial item pool to determine the factor structure and initial psychometric properties of the preliminary IPA (Studies 2 and 3). Our findings offer preliminary evidence for the reliability and validity of the IPA as a novel measure of impostor phenomenon.

The impostor phenomenon, otherwise known as “imposter syndrome,” or the experience of feeling like a “fraud,” has gained increasing attention not only in the popular media but also in the context of achievement-oriented populations including academic and professional settings. Harvey and Katz (1985) operationalized impostor phenomenon as a “psychological pattern rooted in intense, concealed feelings of fraudulence when faced with achievement tasks” (as cited in Hellman & Caselman, 2004, p. 161). People who experience this phenomenon are more likely to doubt their abilities and accomplishments, seeing their own abilities as being incompetent and inferior compared with their peers despite evidence to suggest the contrary (Harvey, 1981; Kolligian & Sternberg, 1991; Langford & Clance, 1993).

Impostor phenomenon has been identified across both men and women (Bussotti, 1990; Langford, 1990; Topping, 1983), across a variety of different cultures (Chae et al., 1995; Clance et al., 1995), and in a wide range of populations including students (Bussotti, 1990; Harvey, 1981; Langford, 1990; Topping, 1983), academic faculty (Hutchins, 2015; Hutchins & Rainbolt, 2017; Topping & Kimmel, 1985), business marketing firms (Fried-Buchalter, 1997; Rohrmann et al., 2016), psychiatrists and doctors (Seritan & Mehta, 2016), and veterans (Stein et al., 2019). This diversity of research has also suggested that nearly 70% of people will experience feelings of being an impostor throughout their life (Gravois, 2007). To demonstrate the commonality of this experience, Kets de Vries (2005) proposed the notion that feeling like an impostor was simply a normal component of human social behavior, whereby people present what they consider to be an acceptable public self, often differing from their private self, with the goal of abiding with social or societal expectations (Cheung, 2018; Kets de Vries, 2005). Despite existing research to suggest that significant feelings of being an impostor are a “normal part” of graduate study (Craddock et al., 2011; Kets de Vries, 2005), students often feel isolated in feeling like an impostor. This has led some to refer to impostor feelings as a “formative” experience in one’s development of their own professional identity (Hutchins & Rainbolt, 2017); however, cross-sectional research has found that these beliefs are associated with feelings of anxiety, depression, self-doubt, and fear of failure (Chrisman et al., 1995; Cokley et al., 2013; Cozzarelli & Major, 1990; Henning et al., 1998; Kumar & Jagacinski, 2006; Leary et al., 2000; Topping, 1983). Limited research has examined long-term outcomes associated with impostor phenomenon.

Furthermore, researchers have investigated a wide range of factors relating to the emergence of impostor phenomenon, including socioeconomic and family (Bussotti, 1990; Clance, 1985; King & Cooley, 1995; Sonnak & Towell, 2001), gender (Badawy et al., 2018; Cusack et al., 2013; Patzak et al., 2017), ethnicity (Ahlfeld, 2009; Ewing et al., 1996; Lige et al., 2017; Peteet et al., 2015), and personality (Bernard et al., 2002; Chae et al., 1995; Clance, 1985; Ferrari & Thompson, 2006; Ross et al., 2001; Thompson et al., 1998; Thompson et al., 2000). Existing research surrounding key features of impostor phenomenon has been primarily observational in nature and based on characteristics outlined by Clance (1985). The initial characteristics outlined by Clance (1985) were described as varying depending on the individual, and additional research has built upon these characteristics to further explore impostors’ external attribution style, self-esteem, personality, and propensity toward perfectionism (Matthew & Clance, 1985; Sakulku & Alexander, 2011). Impostor feelings are often more prominent in transitional situations (e.g., first year of university, first year of graduate studies, and first year of faculty assignment) (Topping & Kimmel, 1985). Feeling like an impostor can elicit beliefs relating to anxiety, self-doubt, and fear of failure (Cokley et al., 2013; Cozzarelli & Major, 1990; Kumar & Jagacinski, 2006; Leary et al., 2000), which aid in undermining individual autonomy, competence, and relatedness (Vaughn et al., 2020). Although Topping and Kimmel (1985) found that feelings of being an impostor decreased when moving beyond transitional stages, they also found that those experiencing impostor phenomenon were less likely to advance in their careers. That is, feelings of being an impostor often accounted for increased career stress, decreased career growth, and decreased aspiration for success (Topping & Kimmel, 1985; Vaughn et al., 2020).

Although some of the core characteristics of impostor phenomenon exist across conceptualizations (e.g., external attribution, low self-esteem, and perfectionism), much of what we know about impostor phenomenon remains uncertain or inconsistent. That is, consistency across conceptualizations of impostor phenomenon is variable, and no existing measurement of impostor phenomenon incorporates all known characteristics into the context of one measurement (i.e., bringing together thoughts, feelings, and behaviors). In addition, since the conception of impostor phenomenon in the 1970s, the academic landscape has evolved significantly in terms of expectations surrounding academic achievement, the need for additional skills (e.g., social media and technology), and the increased competition in the academic culture and subsequent occupational market (e.g., “a bachelor’s degree is the new high school diploma”; Valletta, 2016).

With popular attention of this phenomenon has growing in recent years (see Bravata et al., 2019 for a review), impostor phenomenon has received little empirical attention in terms of its psychometric assessment and the dimensionality of its factors. A recent review of impostor phenomenon assessment (IPA) suggested that current measures are highly inadequate, primarily as a result of their unidimensional scoring of what is otherwise seen as a multidimensional construct (Mak et al., 2019). Notably, many contrasting findings surrounding the relations and factors associated with impostor phenomenon may reflect the inconsistencies in the assessment of these experiences (Bravata et al., 2019). For example, when considering gender, acknowledging that many articles have suggested that women experience significantly higher rates of impostor phenomenon compared with men (e.g., Cusack et al., 2013; Kumar & Jagacinski, 2006), others have found no significant differences across genders (e.g., Cokley et al., 2015; Cromwell et al., 1990; Leonhardt et al., 2017; Rohrmann et al., 2016). In line with these inconsistent findings, Brauer and Proyer (2019) found that gender effects differed depending on context, whereby women experienced higher levels of impostor phenomenon than men in an academic context, but that this difference was not significant within a professional context. These findings were also mirrored by Rohrmann and colleagues (2016), who found no significant gender differences in a population of professionals in leadership positions.

## Existing Measures of Impostor Phenomenon

There is inconsistent validity and reliability across existing measures of impostor phenomenon, as well as a lack of clear multifactor structure reflecting the suggested description in any of the current impostor phenomenon scales (Mak et al., 2019; Topping & Kimmel, 1985). Holmes and colleagues (1993) suggested that many of the discrepancies reported in empirical investigations of impostor phenomenon may be caused by (a) the unclear operationalization of impostor phenomenon in the research literature; (b) the varying methods of measuring impostor phenomenon and identifying impostors based on this unclear definition; (c) the use of varying statistical measurement, including the less preferred median split to classify impostors; and (d) the potential bias for selecting participants from “impostor prone samples.”

### Harvey Impostor Phenomenon Scale (HIPS)

Harvey (1981, 1982) developed a 14-item, 7-point Likert-type scale (0—*not at all true* to 6—*very true)* to identify and measure the self-reported intensity of impostor syndrome in undergraduate and graduate students. Harvey (1981) indicated that the HIPS represented a “homogeneous theoretical construct” that is unidimensional in its assessment. However, factors or at least clusters represented within this measurement are related to self-presentation, self-perception, attributional style, self-esteem, and reinforcing effects of situations. The HIPS is scored using a median split technique, whereby respondents with scores below the sample’s median are classified as being nonimpostors, and those above the median are classified as impostors.

The HIPS has shown inconsistent psychometric properties across studies, ranging from internal consistency of α = .34 to α = .70 (Fujie, 2010; Hellman & Caselman, 2004). Several studies have indicated concerns surrounding content homogeneity and the missing factor structure associated with an overall composite score suggested by the original scoring (Hellman & Caselman, 2004; Mak et al., 2019). Harvey and Katz (1985) proposed that the impostor phenomenon consisted of three core factors: (a) the belief that he or she has fooled other people; (b) the fear of being exposed as an impostor; and (c) the inability to attribute one’s achievement to internal qualities such as ability, intelligence, or skills. However, research has revealed inconsistent results for the factor structure of the HIPS, including a two-factor model (Hellman & Caselman, 2004), a three-factor model (Fried-Buchalter, 1997; Hellman & Caselman, 2004), and a four-factor model (Fried-Buchalter, 1992; Hellman & Caselman, 2004). More recently, Mak et al. (2019) reported a wide range of internal consistency results across five studies using the HIPS, ranging from α =.34 to α = .85. These inconsistent findings suggest insufficient support for the psychometric properties leading some researchers to caution against the use of the HIPS (Hellman & Caselman, 2004).

### Clance Impostor Phenomenon Scale (CIPS)

Building upon clinical observations and responding to criticisms of the HIPS, Clance (1985) developed a 20-item, 5-point Likert-type scale (1—*strongly disagree* to 5—*strongly agree*) designed to assess clinically observed feelings/attributes of impostor phenomenon that were not addressed by the HIPS, including fearing evaluation, feeling less capable than others, and fearing success that could not be repeated (Clance, 1985). The CIPS is also scored using a median split technique for identifying the nonimpostor and impostor groups.

Across 11 studies, internal consistency for the CIPS was strong, ranging from α = .85 to α = .96 (Mak et al., 2019). Several researchers have suggested the presence of a three-factor model of the CIPS (Brauer & Wolf, 2016; French et al., 2008; McElwee & Yurak, 2007): (a) feeling like a fake (α = .84), (b) discounting achievements (α = .73), and (c) attributing success to luck (α = .69). However, there is significant variation in the interpretation of CIPS scores across studies. For example, in some studies, researchers categorize a CIPS score of less than 40 as being indicative of “no impostorism” and ranges of subsequent 10-point score increases as representing “mild,” “moderate,” and “severe” impostor feelings, respectively (Bravata et al., 2019; Clance & O’Toole, 1987). Meanwhile, other studies use the CIPS score to categorically differentiate “nonimpostors” from “impostors” using the median split method. Although the CIPS has demonstrated good internal consistency, evidence for overall construct validity has been mixed. Holmes and colleagues (1993) compared respondent scores on both the CIPS and HIPS with those who they identified as either “nonimpostors” or “impostors” (established through unstructured interviews) in both clinical and nonclinical populations. Their results suggested that the CIPS demonstrated higher sensitivity and reliability when compared to the HIPS, whereby it reduced the incidence of Type 1 (i.e., classifying a nonimpostor as an impostor) and Type 2 (i.e., classifying an impostor as a nonimpostor) errors in cutoff scores. Of course, there may have also been an issue in the “quality and accuracy” of the criterion assessment for classifying the two groups. Despite many of the existing concerns, the CIPS currently represents the most employed measurement of impostor phenomenon in the research literature and in clinical settings (Mak et al., 2019).

### Perceived Fraudulence Scale (PFS)

Following from Kolligian and Sternberg’s (1991) definition of impostor phenomenon as the self-perception of fraudulence in combination with cognitive and affective components, they developed the 51-item PFS. The PFS shares many overlapping factors with the CIPS (Clance, 1985), including fraudulent ideation, self-criticism, achievement pressures, and negative emotions. However, the concept of perceived fraudulence further emphasizes the role of self-worth, impression management, and self-monitoring (Kolligian & Sternberg, 1991). The PFS is identified as the only current measure that considers the multidimensional nature of impostor phenomenon in its theoretical background; however, the scoring of the PFS maintains a unidimensional total score (despite recognition of two underlying factors: inauthenticity and self-deprecation).

The PFS has demonstrated good internal consistency (Kolligian & Sternberg, 1991). Initial validation of the PFS revealed a two-factor model with an overall alpha of 0.94, and factor reliabilities of α = .95 (inauthenticity) and α = .85 (self-deprecation). Given the overlap with factors included in the CIPS, concurrent validity between the CIPS and the PFS is strong (α = .78; Chrisman et al., 1995), representing high intercorrelation (Bernard et al., 2002). However, evidence for internal consistency of the PFS has been mixed, ranging from α = .70 to α = .83 (Kolligian & Sternberg, 1991; Leary et al., 2000). When comparing CIPS and PFS, studies indicate that the brevity of the CIPS allows for greater utility compared with the PFS (Mak et al., 2019). Thus, while Chrisman and colleagues (1995) sought to reduce the PFS from 51 items down to 20 items, mirroring the CIPS, the reliability of the shortened measure decreased to α = .57.

## Summary and Limitations of Existing Scales

At present, the CIPS is the most often employed measure of impostor phenomenon in existing research (Mak et al., 2019); however, this frequency of use does not reflect a higher quality of scale. In fact, much of the multidimensional nature of impostor phenomenon is lost without the examination of subscale scores (Mak et al., 2019). Thus, given the concerns surrounding dimensional clarity, there is still no comprehensive “gold standard” for measuring impostor phenomenon (Mak et al., 2019). Notably, in the absence of a clearly conceptualized theory, the ability to capture the multifaceted nature (i.e., thoughts, feelings, and behaviors) of this experience is greatly limited. To establish a comprehensive, multidimensional measure would further clarify factor structure and foundational characteristics of impostor phenomenon in modern academia, with particular attention to its conceptual clarity and reproducibility across academic samples. In addition, a clear assessment of impostor phenomenon would aid clinicians in identifying its presence, and further specify treatment needs based on the individual target subscales. Thus, in the current research, we sought to develop and clarify a comprehensive conceptualization of impostor phenomenon that incorporates the multidimensional measurement of cognitive, emotional, and behavioral factors, while also seeking to examine potential underlying factors that predict the frequency and intensity of impostor feelings. From this conceptualization, we then sought to develop a psychometrically improved multidimensional assessment of impostor phenomenon through integrating themes present in the research literature.

## Objectives

Given these goals, the objective of this study was to inform a clear theoretical conceptualization of impostor phenomenon based on experiences in an achievement-oriented setting. To do so, we conducted an extensive review of the literature and developed an item pool for a novel IPA (Study 1). We then conducted exploratory factor analyses (Study 1) and confirmatory factor analyses (Study 2) to assess this initial item pool and determine the factor structure and initial psychometric properties (including convergent and divergent validity with self-esteem, perfectionism, and personality) of the preliminary IPA within academic samples (Studies 2 and 3). We also sought to identify between-subjects differences in impostor phenomenon across demographic factors (i.e., gender, age, ethnicity, academic year, and degree); however, given the existing mixed results of these findings (see Bravata et al., 2019 for a review), these investigations were exploratory in nature.

## Study 1

### Item Development and Exploratory Factor Analysis

**Figure 1.**
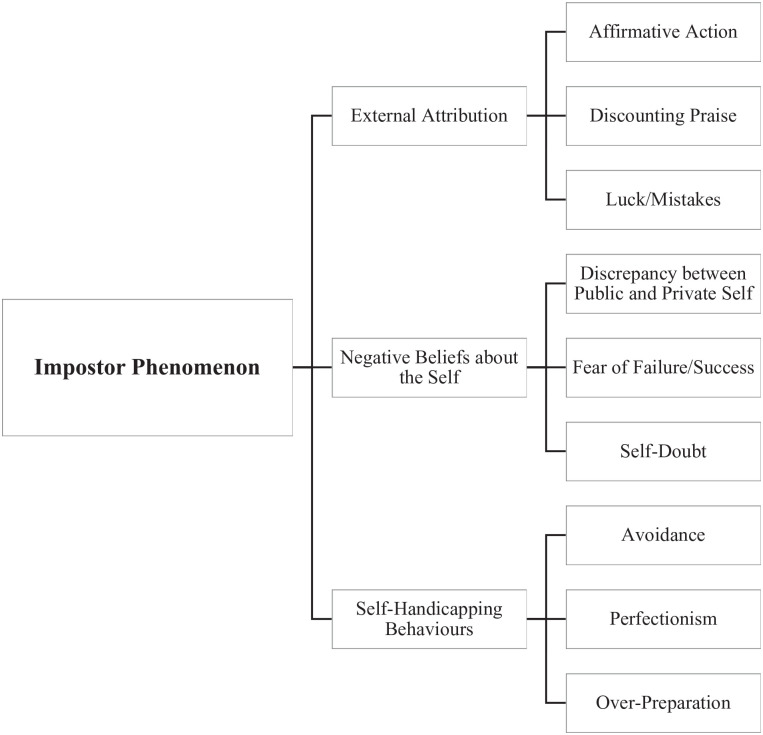
Theoretical Framework for Impostor Phenomenon.

Themes emerging from the existing research literature and measures (i.e., HIPS, CIPS, and PFS) were used to inform model development. Following an extensive review of the literature, we developed a conceptualization of impostor phenomenon as *the subjective experience of perceived self-doubt in one’s abilities and accomplishments compared with others, despite evidence to suggest the contrary*. Within this conceptualization, we developed a theoretical framework with three primary factors: (a) External Attribution, (b) Negative Beliefs About the Self, and (c) Self-Handicapping Behaviors. In addition, within these factors emerged nine subdomains, as presented in Figure 1.

### Participants and Procedure

Given the high prevalence of impostor phenomenon in academia (Craddock et al., 2011; Kets de Vries, 2005), we chose to conduct the initial validation samples within an academic population. A sample of 301 undergraduate students completed an online questionnaire through the university participant pool. Participants who completed the study received 0.5% course credit toward an undergraduate psychology course. Participants’ ages ranged from 17 to 26 years (*M* = 18.27, *SD* = 0.81), and 71% of the population self-identified as women. Participants represented a moderately diverse range of ethnic origins (43.9% Caucasian, 35.9% Asian, 8% Other, 8% Middle Eastern, 3.7% Black, and 0.3% Indigenous).

### Measures

#### Impostor Phenomenon

Following the development of the theoretical framework, we employed deductive methods (i.e., literature review and existing measures) to generate items with the objective of creating an updated and multidimensional model of impostor phenomenon. Items included those adapted from existing measures of impostor phenomenon, as well as those which we created and adapted in line with the above theoretical framework. An initial item pool of 81 items was subjected to rigorous psychometric refinement and several subject matter expert reviews. To assess content validity of the novel measure, we consulted a panel of 12 graduate students in the department of psychology to provide feedback regarding the initial items at face validity. This panel of graduate students was representative of the population of interest and had strong theoretical understanding of test construction processes. The panel had the opportunity to provide written feedback regarding the “fit” of items to adequately capture experiences of impostor phenomenon based on our conceptualization, and subsequently categorized items into an open number of factors based on the emergence of common themes. The suggested factors closely mirrored the proposed theoretical framework. From this feedback, the item pool was modified to remove redundant and unclear items (*n* = 9), leaving 72 items falling into three factors: (a) External Attribution, (b) Negative Beliefs About the Self, and (c) Self-Handicapping Behaviors (see Table 1). Impostor phenomenon was then measured using the preliminary 72-item IPA (see Table 2). The creation of this new measure was guided by test construction principles outlined by DeVellis (2017) including (a) Clearly determining what you want to measure, (b) generating an item pool, (c) determining the format for measurement, (d) Consulting experts to review the item pool, (e) considering inclusion of validation items, (f) administering items to a development sample, (g) evaluating the items, and (h) optimizing scale length (Devellis, 2017). Participants responded to items on a 6-point Likert-type scale, from 1 (*strongly disagree*) to 6 (*strongly agree*). A six-item scale was determined to capture the dimensional nature of this construct. This decision was grounded in previous research to suggest a 6-point scale as optimal in self-report assessments (Preston & Colman, 2000). Overall, impostor phenomenon, as well as each of the individual subscales, is calculated by obtaining the mean of all items, such that a higher overall score is indicative of higher levels of impostor phenomenon.

**Table 1.** Initial Item Pool Factors.

Factor	Number of items
External Attribution	17
Discounting Praise	9
Luck/Mistakes	6
Affirmative Action	2
Negative Beliefs About the Self	36
Fear of Failure/Success	10
Discrepancy of Private/Public Self	18
Self-Doubt	8
Self-Handicapping Behaviors	20
Perfectionism	5
Overpreparation	7
Avoidance	7

**Table 2:** IPA Initial Item Set.

Item	Subdomain
External Attribution	
1. I obtained my present position because of something about me that I didn’t work for (e.g., coming from an underrepresented group).	Affirmative Action
2. I obtained my present position solely because of an affirmative action policy.	Affirmative Action
3. I feel I deserve whatever honors, recognition, or praise I receive. (r)	Discounting Praise
4. I find it easy to accept compliments about my intelligence. (r)	Discounting Praise
5. I often feel I receive praise or grades that I don’t deserve.	Discounting Praise
6. If I receive a great deal of praise and recognition for something I’ve accomplished, I tend to discount the importance of what I’ve done.	Discounting Praise
7. It’s hard for me to accept compliments or praise about my intelligence or accomplishments.	Discounting Praise
8. On some occasions when someone has praised me for something, I tend to feel that I fooled them.	Discounting Praise
9. When I receive a compliment about my academic or professional abilities, I sometimes find myself making excuses for explaining away the compliment.	Discounting Praise
10. When I receive a compliment, I find it difficult to accept the compliment, and often explain it away or give credit to others.	Discounting Praise
11. It is easy for me to give myself credit for the good things that happen to me, professionally or socially. (r)	Discounting Praise
12. I feel that I have attained my present academic or professional position through “pulling strings” or “having connections.”	Luck/Mistake
13. I often feel that my success has been due to some kind of luck.	Luck/Mistake
14. At times, I have felt I am in my present position or academic program through some kind of mistake.	Luck/Mistake
15. I sometimes think I obtained my present position or gained my present success because I happened to be in the right place at the right time or knew the right people.	Luck/Mistake
16. My achievements have been due more to external factors, such as luck or effort, rather than to my own inherent abilities.	Luck/Mistake
17. Sometimes, I believe that my success in my life or in my job has been the result of some kind of error.	Luck/Mistake
Negative Beliefs About Self	
18. I can give the impression that I’m more competent than I really am.	Discrepancy
19. I feel that there is a significant disparity between the “intellectual self” that others perceive and the “intellectual self” that I really am.	Discrepancy
20. I have sometimes convinced an important person that I am brighter or more talented than I really am.	Discrepancy
21. I often feel I am concealing secrets about my abilities from others.	Discrepancy
22. I often worry about not succeeding on a task, even though others around me have considerable confidence that I will do well.	Discrepancy
23. I sometimes feel there’s something false or misleading about me that others don’t notice.	Discrepancy
24. I would describe myself as an “authentic” person. (r)	Discrepancy
25. I’m afraid people important to me may find out that I’m not as capable as they think I am.	Discrepancy
26. If I get a high grade on a work assignment, I tend to feel that I’ve fooled my teacher or supervisor.	Discrepancy
27. In general, I act more competently than I feel that I really am.	Discrepancy
28. People tend to believe I am more competent than I really am.	Discrepancy
29. Significant people in my life tend to believe that I am more academically or professionally competent than I really am.	Discrepancy
30. In some situations, I feel like a “great pretender”: that is, I’m not as genuine as others think I am.	Discrepancy
31. My private feelings and perceptions about myself sometimes conflict with the impressions I give others through how I act.	Discrepancy
32. My public and private self are the same person. (r)	Discrepancy
33. Sometimes, I am afraid I will be discovered for who I really am.	Discrepancy
34. Sometimes. I’m afraid others will discover how much knowledge or ability I really lack.	Discrepancy
35. At a social event, I sometimes feel that I try to impress people by acting more intelligently than I really feel I am.	Discrepancy
36. I feel confident that I will succeed in the future. (r)	Fear of Failure/Success
37. I often foresee failure when entering new situations that require a demonstration of my abilities.	Fear of Failure/Success
38. I often achieve success on a project or test when I have anticipated that I would fail.	Fear of Failure/Success
39. I tend to remember the incidents where I have not done my best more than those times that I have done my best.	Fear of Failure/Success
40. I’m often afraid that I will fail at a new assignment or undertaking even though I generally do well at what I attempt.	Fear of Failure/Success
41. If I’m going to receive a promotion or gain recognition of some kind, I hesitate to tell others until it is an accomplished fact.	Fear of Failure/Success
42. When I am about to take on a new and challenging project, task, or responsibility, I am more inclined to remember my past successes rather than my past failures. (r)	Fear of Failure/Success
43. When I’m praised for something, I sometimes wonder if I will be able to do as well the next time.	Fear of Failure/Success
44. When I’ve succeeded at something and received recognition for my accomplishments, I have doubts that I can keep repeating that success.	Fear of Failure/Success
45. When people praise me for something I’ve accomplished, I’m afraid I won’t be able to live up to their expectations of me in the future.	Fear of Failure/Success
46. Even though I feel that I have a lot of potential, I sometimes feel like an intellectual “fraud” or “phony.”	Self-Doubt
47. I consider my accomplishments adequate for this stage in my life. (r)	Self-Doubt
48. I have often succeeded on a test or task even though I was afraid that I would not do well before I undertook the task.	Self-Doubt
49. I often feel that I am “in over my head” or beyond my capabilities in my area of work or study.	Self-Doubt
50. I often worry about whether others will view me as a success or a failure.	Self-Doubt
51. Even in situations for which I am well-prepared (e.g., studied very hard and long for an examination or worked tirelessly on a project), I still have doubts about my ability to perform well.	Self-Doubt
52. I am often surprised when I perform well on a project or a test.	Self-Doubt
53. I often compare my ability to those around me and believe they are more intelligent than I am.	Self-Doubt
Self-Handicapping Behaviors	
54. I avoid evaluations if possible and have a dread of others evaluating me.	Avoidance
55. I worry about my ability to complete a task and often end up delaying making decisions about the task until it is too late.	Avoidance
56. I find myself often leaving tasks to the last minute.	Avoidance
57. I put off making decisions out of fear that I won’t make the right one.	Avoidance
58. I try not to get too involved in competitive environments, so it won’t hurt so much if I lose or do poorly.	Avoidance
59. I typically delay getting started on tasks because I worry that I’m not up to the challenge.	Avoidance
60. In preparing for deadlines, I often waste time doing other things.	Avoidance
61. I become very invested in my assigned tasks and find it difficult to focus on anything else.	Overpreparation
62. I often feel like I have to put more effort into my tasks because I am not as smart as those around me.	Overpreparation
63. I often feel that I have to work harder than others to achieve all that I do.	Overpreparation
64. I often find myself putting more effort into tasks compared with others.	Overpreparation
65. I often find myself spending more time than necessary in completing tasks or assignments.	Overpreparation
66. I often tell others that I studied or worked less (i.e., spent less time) on a professional/intellectual project than I actually did.	Overpreparation
67. Others have told me that I often do more than necessary when it comes to completing a task.	Overpreparation
68. I feel discouraged if I’m not “the best” or at least “very special” in situations that involve achievement.	Perfectionism
69. I often get “down on myself” when I perform less than perfectly on a task or a problem.	Perfectionism
70. I rarely do a project or task as well as I’d like to do it.	Perfectionism
71. Mostly, I find that I measure up to the standards that I set for myself. (r)	Perfectionism
72. When it comes to achieving and attaining goals, I suppose one might call me a “perfectionist.”	Perfectionism

*Note.* “(r)” indicates a reverse-scored item.

### Eternal Attribution (17 Items)

A consistent theme in impostor phenomenon is externalizing positive events and viewing them as temporary, while internalizing and generalizing negative events. Early research by Clance and Imes (1978) suggested that individuals with impostor phenomenon commonly denied their personal competence in addition to discounting praise from others. This includes significant difficulties internalizing success and accepting praise as being valid or true. Rather, those with impostor phenomenon discount positive feedback despite objective evidence to suggest successful achievements, instead attributing success to external factors (Chae et al., 1995; Harvey, 1981; Thompson et al., 1998; Topping & Kimmel, 1985). Attributing successes to luck or mistakes, rather than to their “true abilities” is what researchers typically describe as being characteristic of impostor phenomenon (e.g., Edwards et al., 1987). Those with impostor phenomenon view this feedback as being incongruent with their personal perceptions of achieved success, and instead hold the belief that they are “not deserving” of their accomplishments (Casselman, 1991; Edwards et al., 1987; Sakulku & Alexander, 2011). This leads to an unwillingness to accept compliments or praise relating to accomplishments.

In addition, to incorporate consideration of cultural factors, including the presence of minority stress, cultural expectations, and “survivor’s guilt” (e.g., Austin et al., 2009), we included two items assessing perceived views toward the role of affirmative action in current academic position. This factor incorporates societal-level considerations of the way an individual perceives their role based on social hierarchy (e.g., Feenstra et al., 2020). Altogether, the first factor (External Attribution) is cognitive in nature and represents a significant effort to diminish one’s achievements. Within this theme, we included three subdomains: discounting praise (nine items; e.g., “I often feel I receive praise or grades that I don’t deserve”), luck/mistakes (six items; e.g., “At times, I have felt I am in my present position or academic program through some kind of mistake”), and affirmative action (two items; e.g., “I obtained my present position because of something about me that I didn’t work for (e.g., coming from an underrepresented group)”).

### Negative Beliefs About the Self (36 Items)

Fear and guilt surrounding failure is one of the key features of impostor phenomenon (Clance, 1985; Kets de Vries, 2005). Those with impostor phenomenon commonly focus attention on evidence suggesting that they do not deserve recognition for their achievements, even if this evidence is limited (Clance, 1985). They demonstrate a significant gap in the emotional assessment and appraisal of their own abilities, particularly when compared to their actual, objective, output (Want & Kleitman, 2006). Thus, the second subdomain taps into low self-confidence relating to one’s own competence. Given the disproportionate standards that those with impostor phenomenon set for themselves, along with their lack of confidence in their future self to meet those standards (Edwards et al., 1987), they are commonly left feeling overwhelmed, and overgeneralize perceived failures when eventually they are not able to achieve such high standards. In addition, in the presence of mistakes, or when they feel that they did not perform to their highest standards, those with impostor phenomenon experience significant feelings of shame and humiliation, which act to further reinforce their self-doubt and fears of future failures (and successes; Clance, 1985).

People with impostor phenomenon demonstrate discrepant and low appraisals of their performance outcomes (Want & Kleitman, 2006). That is, they are more likely to perceive a “gap” between how they view their performance and how others view their achievements. This maps onto the fear of being discovered, or the perception of oneself as “phony” (Edwards et al., 1987), suggesting negative beliefs and fears of others discovering their perceived incompetence. Thus, the second factor (negative beliefs about self) represents emotional experiences associated with feelings of fear, guilt, and shame (Clance & Imes, 1978). In line with this theme are three subdomains: fear of success/failure (10 items; e.g., “When I’m praised for something, I sometimes wonder if I will be able to do as well the next time”), discrepancy between the public and private self (18 items; e.g., “Sometimes I’m afraid others will discover how much knowledge or ability I really lack”), and self-doubt (eight items; e.g., “I often feel that I am “in over my head” or beyond my capabilities in my area of work or study”).

### Self-Handicapping Behaviors (19 Items)

Impostor phenomenon has also been associated with behavioral responses (e.g., Lane, 2015) that are not otherwise captured in the existing measurement scales. For example, there are significant patterns of self-handicapping behaviors associated with impostor phenomenon (Cowman & Ferrari, 2002; Ferrari & Thompson, 2006; Ross et al., 2001). These behaviors are represented in the second level of the Impostor Cycle, whereby anxiety, self-doubt, and worry prompt a behavioral response (i.e., overpreparation and procrastination; Clance, 1985). Self-handicapping is defined as a group of self-deprecating behaviors that an individual engages in as a manner of protecting their personal self-image or self-esteem (Leary et al., 2000; Want & Kleitman, 2006). Self-handicapping represents a self-presentational strategy whereby downplaying one’s achievements functions as a strategy to avoid negative interpersonal implications associated with potential failure and negative evaluation (Ferrari & Thompson, 2006; Leary et al., 2000). People who engage in self-handicapping behaviors intentionally introduce an obstacle that is within their control (as a “handicap”) to impede chances of success or progress toward a goal, allowing potential failures to be attributed to this handicap, rather than to themselves (Ferrari & Thompson, 2006; Strube, 1986; Want & Kleitman, 2006). Impostor phenomenon is associated with the desire to portray an image of being a “super-person,” otherwise seen as perfectionistic cognitions (Clance & Imes, 1978; Ferrari & Thompson, 2006), which elicit behaviors in attempt to outperform peers as one way of compensating for feelings of self-doubt. These self-handicapping behaviors are typically associated with perfectionism, overpreparation, and avoidance that further enhance individuals’ perceived inadequacy (Clance & Imes, 1978; Edwards et al., 1987). Impostor phenomenon also perpetuates procrastination behaviors out of an effort to avoid or delay the potential for outcomes that may be less than their ideal standard of success (Ferrari & Thompson, 2006).

Although those with impostor phenomenon may recognize this pattern of self-handicapping behaviors, they often hold the belief that without this approach to work, they would encounter failure (Clance, 1985). However, existing measures of impostor phenomenon typically exclude behavioral components (i.e., what actions are people taking as a result of these thoughts and emotions?). Thus, for the third subdomain (self-handicapping behaviors), we accounted for behaviors including perfectionism (five items; e.g., “I rarely do a project or task as well as I’d like to do it”), overpreparation (seven items; “I often find myself putting more effort into tasks compared to others”), and avoidance (seven items; e.g., “I avoid evaluations if possible and have a dread of others evaluating me”). This behavioral piece is a relatively novel inclusion in the assessment of impostor phenomenon, as previous scales have focused primarily on cognitive and emotional perceptions of the self (e.g., “I feel like a fraud”), rather than identifying behaviors associated with impostor phenomenon.

### Study 1 Results

#### Exploratory Factor Analyses

We conducted exploratory factor analyses using SPSS Version 26.0 (IBM Corp., 2019) to determine the optimal factor structure to fit the initial IPA data. The Kaiser–Meyer–Olkin measure of sampling adequacy was .91, above the minimum recommended value of .60 (Kaiser, 1974), and Bartlett’s test of sphericity was significant, χ2(2556) = 11,115, *p* < .001. Taken together, the results of these tests suggested that the current data were suitable for subsequent factor analysis. We then computed interitem correlations and analyzed the resulting correlation matrix with principal axis factoring extraction. We determined the number of factors to extract by considering existing theoretical modeling, Kaiser’s eigenvalue criterion, and the scree plot, which all suggested a three- or four-factor solution.

Given the current multidimensional conceptualization of impostor phenomenon, the various dimensions were assumed to be nonorthogonal, and thus, we employed an oblique rotation. We tested both the hypothesized three-factor structure (as defined by the scale facets) and the four-factor structure using a promax rotation. A three-factor solution explained 37.3% of the total variance. A four-factor solution explained 40.2% of the variance; however, given the small increase in variance (~3%), increase in cross-loadings, and conceptual similarities, we selected the three-factor solution as the most conceptually and empirically parsimonious structure.

Seventeen items (Items 3, 4, 6, 7, 11, 18, 24, 27, 31, 34, 36, 42, 47, 49, 57, 61, and 66) were removed as they did not contribute to the simple factor structure and failed to meet the minimum criteria (i.e., loadings less than 0.32 were excluded as they were not considered to be substantial; Comrey & Lee, 1992). Following removal of these items, we conducted a second exploratory factor analysis, and the 55 remaining items loaded cleanly and substantially onto the three factors. A three-factor structure remained the best fit for the data, accounting for 40.0% of the variance.

#### Principal Components Analyses

For the final stage, we conducted a principal components analysis of the remaining 55 items, using promax and oblimin rotations using SPSS Version 26.0 (IBM Corp., 2019). A promax rotation provided the best-defined factor structure. One item had a cross-loading above .32 (Item 48), and low loading on its primary loading, and was thus removed. Five items had a cross-loading above .32 (Items 1, 12, 64, 67, and 72), but demonstrated strong factor loadings on their primary factor (i.e., above .60), and were thus retained. The factor loading matrix for this final 54-item solution is presented in Table 3, with the final factor structure accounting for 43.6% of the total variance.

**Table 3.** Pattern Matrix Factor Loadings for the Three-Factor Model Using Principal Axis Factoring With Promax Rotation.

	Factor			Uniqueness
Variable	DA	PD	SHB	
51. Even in situations for which I am well-prepared (e.g., studied very hard and long for an examination or worked tirelessly on a project), I still have doubts about my ability to perform well.	**0.830**			0.401
63. I often feel that I have to work harder than others to achieve all that I do.	**0.777**			0.505
69. I often get “down on myself” when I perform less than perfectly on a task or a problem.	**0.747**			0.546
22. I often worry about not succeeding on a task, even though others around me have considerable confidence that I will do well.	**0.743**			0.515
64. I often find myself putting more effort into tasks compared with others.	**0.741**		−0.487	0.527
72. When it comes to achieving and attaining goals, I suppose one might call me a “perfectionist.”	**0.733**		−0.421	0.560
67. Others have told me that I often do more than necessary when it comes to completing a task.	**0.690**		−0.609	0.507
53. I often compare my ability to those around me and believe they are more intelligent than I am.	**0.683**			0.527
45. When people praise me for something I’ve accomplished, I’m afraid I won’t be able to live up to their expectations of me in the future.	**0.675**			0.401
44. When I’ve succeeded at something and received recognition for my accomplishments, I have doubts that I can keep repeating that success.	**0.675**			0.426
62. I often feel like I have to put more effort into my tasks because I am not as smart as those around me.	**0.674**			0.550
40. I’m often afraid that I will fail at a new assignment or undertaking even though I generally do well at what I attempt.	**0.647**			0.582
65. I often find myself spending more time than necessary in completing tasks or assignments.	**0.642**			0.646
43. When I’m praised for something, I sometimes wonder if I will be able to do as well the next time.	**0.629**			0.545
68. I feel discouraged if I’m not “the best” or at least “very special” in situations that involve achievement.	**0.587**			0.680
50. I often worry about whether others will view me as a success or a failure.	**0.560**			0.560
39. I tend to remember the incidents where I have not done my best more than those times that I have done my best.	**0.527**			0.688
37. I often foresee failure when entering new situations that require a demonstration of my abilities.	**0.497**			0.498
54. I avoid evaluations if possible and have a dread of others evaluating me.	**0.493**			0.542
58. I try not to get too involved in competitive environments, so it won’t hurt so much if I lose or do poorly.	**0.479**			0.707
52. I am often surprised when I perform well on a project or a test.	**0.467**			0.569
10. When I receive a compliment, I find it difficult to accept the compliment, and often explain it away or give credit to others.	**0.451**			0.756
9. When I receive a compliment about my academic or professional abilities, I sometimes find myself making excuses for explaining away the compliment.	**0.448**			0.646
25. I’m afraid people important to me may find out that I’m not as capable as they think I am.	**0.444**			0.524
46. Even though I feel that I have a lot of potential, I sometimes feel like an intellectual “fraud” or “phony.”	**0.437**			0.445
41. If I’m going to receive a promotion or gain recognition of some kind, I hesitate to tell others until it is an accomplished fact.	**0.434**			0.854
38. I often achieve success on a project or test when I have anticipated that I would fail.	**0.367**			0.835
**12. I feel** that I have attained my present academic or professional position through “pulling strings” or “having connections.”		**0.925**	−0.367	0.436
15. I sometimes think I obtained my present position or gained my present success because I happened to be in the right place at the right time or knew the right people.		**0.795**		0.571
5. I often feel I receive praise or grades that I don’t deserve.		**0.762**		0.477
26. If I get a high grade on a work assignment, I tend to feel that I’ve fooled my teacher or supervisor.		**0.730**		0.463
1. I obtained my present position because of something about me that I didn’t work for (e.g., coming from an underrepresented group).	−0.352	**0.712**		0.639
17. Sometimes I believe that my success in my life or in my job has been the result of some kind of error.		**0.689**		0.409
14. At times, I have felt I am in my present position or academic program through some kind of mistake.		**0.683**		0.501
13. I often feel that my success has been due to some kind of luck.		**0.678**		0.522
16. My achievements have been due more to external factors, such as luck or effort, rather than to my own inherent abilities.		**0.672**		0.545
8. On some occasions when someone has praised me for something, I tend to feel that I fooled them.		**0.634**		0.410
30. In some situations, I feel like a “great pretender”: that is, I’m not as genuine as others think I am.		**0.582**		0.474
33. Sometimes, I am afraid I will be discovered for who I really am.		**0.565**		0.452
2. I obtained my present position solely because of an affirmative action policy.		**0.512**		0.822
21. I often feel I am concealing secrets about my abilities from others.		**0.469**		0.677
23. I sometimes feel there’s something false or misleading about me that others don’t notice.		**0.445**		0.533
19. I feel that there is a significant disparity between the “intellectual self” that others perceive and the “intellectual self” that I really am.		**0.426**		0.625
20. I have sometimes convinced an important person that I am brighter or more talented than I really am.		**0.404**		0.705
28. People tend to believe I am more competent than I really am.		**0.400**		0.625
35. At a social event, I sometimes feel that I try to impress people by acting more intelligently than I really feel I am.		**0.394**		0.724
29. Significant people in my life tend to believe that I am more academically or professionally competent than I really am.		**0.383**		0.563
56. I find myself often leaving tasks to the last minute.			**0.898**	0.351
60. In preparing for deadlines, I often waste time doing other things.			**0.892**	0.373
59. I typically delay getting started on tasks because I worry that I’m not up to the challenge.			**0.691**	0.421
55. I worry about my ability to complete a task, and often end up delaying making decisions about the task until it is too late.			**0.637**	0.408
71. Mostly, I find that I measure up to the standards that I set for myself. (r)			**0.488**	0.683
32. My public and private self are the same person. (r)			**0.457**	0.767
70. I rarely do a project or task as well as I’d like to do it.			**0.365**	0.736

*Note.* Values below 0.32 are suppressed, bolded values represent loadings > 0.32 DA = Doubts About Achievement (27 items); PD = Personal Discrepancy (20 items); SHB = Self-Handicapping Behaviors (seven items).

**Figure 2.**
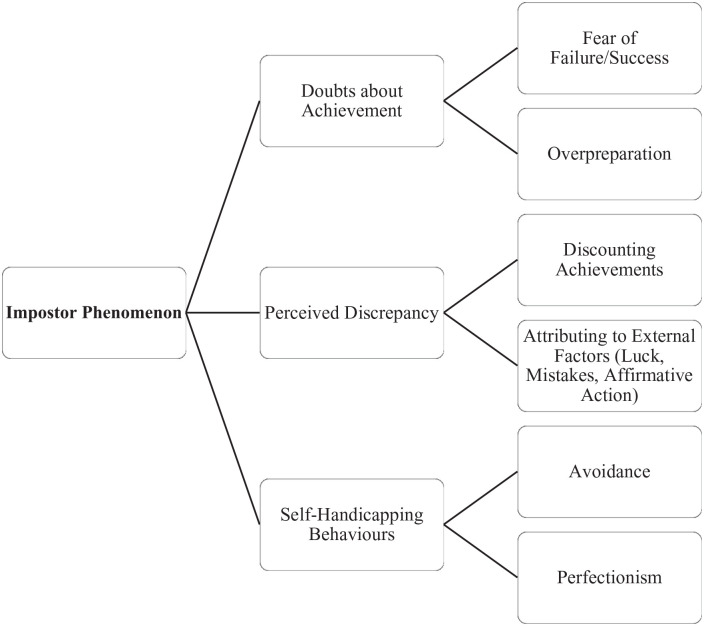
Revised Theoretical Framework for Impostor Phenomenon Based on Exploratory Factor Analysis.

**Figure 3.**
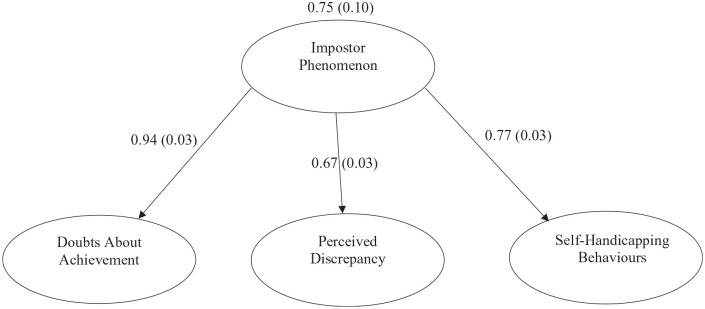
Model Fit for Imposter Phenomenon Assessment Based on Confirmatory Factor Analysis.

Based on the theoretical background and analysis of item loadings, we relabeled the three factors: (a) Doubts About Achievement (27 items), (b) Perceived Discrepancy (20 items), and (c) Self-Handicapping Behaviors (seven items). See Figure 2 for an updated framework and Figure 3 for model fit statistics. All factors correlated in the expected direction and were significantly positively correlated with one another (see Table 4). Internal consistency of the 54-item scale was excellent (α = .95), and subscales representing the three factors also demonstrated strong internal consistency (α = .93, α = .92, and α = .81, respectively). Skewness and kurtosis values for scores on the total 54-item measure and each subscale were in the acceptable range (see Table 5).

**Table 4.** Descriptive Statistics and Bivariate Correlation Matrix for Study 1 IPA Total and Subscales.

Variable	*M*	*SD*	α	1	2	3	4
1. DA	4.15	0.81	.93	—	—	—	—
2. PD	3.01	0.83	.92	**.50**	—	—	—
3. SHB	3.65	0.76	.81	**.43**	**.51**	—	—
4. Total IPA	3.66	0.67	.95	**.88**	**.83**	**.66**	—

*Note. N* = 283. Bold indicates significance at *p* < .01; two-tailed test. IPA = Impostor Phenomenon Assessment; DA = Doubts About Achievement; PD = Personal Discrepancy; SHB = Self-Handicapping Behaviors.

**Table 5.** Skewness and Kurtosis of IPA Total and Subscales.

Variable	Skewness	*SE*	Kurtosis	*SE*
DA	−.249	.145	−.500	.289
PD	.281	.145	−.369	.289
SHB	−.158	.145	−.462	.289
Total IPA	.035	.145	−.332	.289

*Note. N* = 283. IPA = Impostor Phenomenon Assessment; DA = Doubts About Achievement; PD = Personal Discrepancy; SHB = Self-Handicapping Behaviors.

### Study 1 Discussion

Through panel review and exploratory factor analysis, a three-factor structure emerged for a novel 54-item IPA: (a) Doubts About Achievement, (b) Perceived Discrepancy, and (c) Self-Handicapping Behaviors. The initial scale demonstrated excellent initial psychometric properties.

In comparing the factor structure emerging from the data to the initial proposed framework, it is notable that the factor structure highly resembles the proposed theoretical framework, with particular attention to similarities with Factor 2 (Negative Beliefs About the Self—renamed Perceived Discrepancy) and Factor 3 (Self-Handicapping Behaviors). Factor 2 was renamed Perceived Discrepancy because of the notable difference where items associated with “fear of failure and success” loaded primarily onto Factor 1. Factor 1 emerged as the most variable within the proposed factor structure, incorporating items relating to subdomains including “discounting” and “perfectionism”; however, the main theme of these items represented links with achievement and performance (e.g., “When I receive a compliment about my academic or professional abilities, I sometimes find myself making excuses for explaining away the compliment”; “I often get “down on myself” when I perform less than perfectly on a task or a problem). As such, Factor 1 was relabeled from External Attribution, which is now primarily accounted for in Factor 2, to Doubts about Achievement.

## Study 2

### Confirmatory Factor Analysis

Following the initial factor structure emerging in Study 1, we conducted confirmatory factor analyses to replicate the factor structure and initial psychometric properties of the preliminary IPA in an independent sample of undergraduate students.

### Participants and Procedure

Mirroring Study 1, a sample of 589 undergraduate students completed an online questionnaire through the university participant pool and received course credit toward an undergraduate psychology course. Participants’ ages ranged from 18 to 41 years (*M* = 19.17, *SD* = 1.62), 66% of the population self-identified as women, and participants represented a moderately diverse range of ethnic origins (44.3% Caucasian, 37.7% Asian, 8.8% Other, 5.9% Middle Eastern, 2.5% Black, and 0.7% Indigenous). Data for 35 participants were removed due to incomplete and inattentive responding, leaving a final sample of 554 participants for analyses.

### Measures

Participants completed the updated 54-item IPA to assess impostor phenomenon. Mirroring the initial item set, participants responded to items on a 6-point Likert-type scale, from 1 (*strongly disagree*) to 6 (*strongly agree*). We calculated the overall mean for impostor phenomenon such that a higher overall score was indicative of higher levels of impostor phenomenon. In addition to the overall score, we calculated the mean of items from each subscale to provide scores on each of the three individual factors (Doubts About Achievement, Perceived Discrepancy, and Self-Handicapping Behaviors).

### Study 2 Results

Descriptive statistics and bivariate correlations for the total IPA score and three subscales are presented in Table 6. Internal consistency for the IPA Total and its three subscales was excellent (see Table 6).

**Table 6:** Descriptive Statistics and Bivariate Correlation Matrix for Study 2 IPA Total and Subscales.

Variable	*M*	*SD*	α	1	2	3	4
1. DA	3.93	0.82	.93	—	—	—	—
2. PD	3.01	0.84	.92	**.55**	—	—	—
3. SHB	3.96	0.85	.73	**.55**	**.41**	—	—
4. Total IPA	3.59	0.70	.95	**.91**	**.83**	**.66**	—

*Note. N* = 554. Bold indicates significant at *p* < .01; two-tailed test. IPA = Impostor Phenomenon Assessment; DA = Doubts About Achievement; PD = Personal Discrepancy; SHB = Self-Handicapping Behaviors.

#### Confirmatory Factor Analysis

To assess the initial factor structure, we conducted confirmatory factor analysis and structural equation modeling using Mplus version 8 (Muthén & Muthén, 1998-2012). The fit statistics supported a three-factor model, χ2(1374). = 5,064, *p < .*001. Findings suggested adequate fit reflected in the root-mean-square error of approximation (RMSEA = 0.07, 90% CI = [.069, .072], *p <* .001) and the standardized root-mean-square residual (SRMR = 0.08; Hu & Bentler, 1999; MacCallum et al., 1996). In addition, all loadings were significant (*p <.* 01). Although some fit statistics suggested moderate fit (Tucker-Lewis Index (TLI) = 0.72, Comparative Fit Index (CFI) = 0.73), it is possible that this is due to the nature of the current measurement as a hierarchical restricted model with multiple dimensions, as well as the large number of items.

#### Exploratory Analyses

To assess for differences in gender, we conducted an independent samples t-test. Results suggested significant differences between men and women on all factors and total impostor phenomenon (marginally significant for Self-Handicapping Behaviors; Table 7). To assess for differences across ethnicity and age, we conducted a one-way analysis of variance (ANOVA). Results suggested significant differences in total impostor phenomenon across ethnicity, *F*(5, 548) = 3.56, *p* < .01; Table 8. Post hoc comparison revealed a significant mean difference in impostor phenomenon between Asian students and Caucasian students (*p <* .01). However, we interpret these findings with caution given the limited diversity of the current sample. There were no significant differences in total impostor phenomenon across age, *F*(9, 504) = 0.88, *p =* .54.

**Table 7:** Differences Between Genders in Study 2.

Variable	Men (*n* = 186) *M* (*SD*)	Women (*n* = 366) *M* (*SD*)	*t*	*p*
DA	3.57 (0.76)	4.12 (0.79)	−7.84	.00**
PD	2.90 (0.85)	3.06 (0.83)	−2.17	.03*
SHB	3.87 (0.76)	4.01 (0.89)	−1.95	.05
Total IPA	3.36 (0.69)	3.71 (0.68)	−5.76	.00**

*Note.* IPA = Impostor Phenomenon Assessment; DA = Doubts About Achievement, PD = Perceived Discrepancy, SHB = Self-Handicapping Behaviors.

* *p* < .05. **p < .01.

**Table 8:** Descriptive Statistics for Ethnicity and Impostor Phenomenon in Study 2.

Ethnicity	*n*	*M* (*SD*)
Caucasian	252	3.48 (0.73)
Asian	208	3.73 (0.63)
Middle Eastern	33	3.62 (0.68)
Black	12	3.58 (0.79)
Indigenous	4	4.19 (0.34)
Other	46	3.58 (0.78)

## Study 3

### Confirmatory Factor Analysis and Psychometric Validation

Following the confirmed factor structure in Studies 1 and 2, we conducted further confirmatory factor analyses and assessment of convergent and divergent validity to replicate the factor structure and initial psychometric properties of the preliminary IPA.

### Participants and Procedure

Mirroring Studies 1 and 2, a sample of 785 undergraduate and graduate students consented to complete the online questionnaire through the university participant pool and mass email recruitment. Eligible undergraduate student participants received course credit toward an undergraduate psychology course. Data were removed for 74 participants who consented to participate and did not complete any additional components of the study, and for 138 participants who completed less than 85% of the study. An additional 14 participants were removed due to inattentive responding (i.e., failing at least 50% of attention checks). Thus, our final sample for analyses consisted of 562 students.

Participants’ ages ranged from 17 to 69 years (*M* = 20.23, *SD* = 5.41), 69% of the population self-identified as women (27% men, 2% Trans, and 1% nonbinary), and participants represented a moderately diverse range of ethnic origins (45.0% Caucasian, 37.6% Asian, 8.1% Other, 6.6% Middle Eastern, 2.3% Black, and 0.4% Indigenous). Undergraduate students comprised 87.6% of the sample (*n =* 481), with graduate students representing 12.4% of the sample (*n =* 68). To assess objective academic success, we also asked participants to provide their approximate grade point average (GPA) in percentage (range = 60%–100%, *M =* 85.84%, *SD =* 7.52%).

### Measures

#### Impostor Phenomenon

Participants completed the updated 54-item IPA. responding to items on a 6-point Likert-type scale, from 1 (*strongly disagree*) to 6 (*strongly agree*). We then calculated an overall mean impostor phenomenon score in addition to mean scores on each of the three individual subscales as suggested in Study 1, such that a higher overall score was indicative of higher levels of impostor phenomenon. In addition to the overall score, we calculated scores on each of the three individual subscales by obtaining the mean of items within the respective subscale (Doubts About Achievement, Perceived Discrepancy, and Self-Handicapping Behaviors).

#### Personality

To assess personality, we used the 10-item Big Five Inventory (BFI-10; Rammstedt & John, 2007). Participants responded to items on a 5-point Likert-type scale from 1 (*disagree strongly*) to 5 (*agree strongly*), representing extraversion (e.g., “*I see myself as someone who is outgoing, sociable”*), agreeableness (e.g., “*I see myself as someone who is generally trusting”)*, conscientiousness (e.g., “*I see myself as someone who does a thorough job”*), neuroticism (e.g., “*I see myself as someone who gets nervous easily”*), and openness (e.g., “*I see myself as someone who has an active imagination*”). Scores on each of the subscales were calculated by obtaining a sum of items on each respective subscale.

#### Perfectionism

To assess perfectionism, we used the 16-item Big Three Perfectionism Scale—Short Form (BTPS-SF; Feher et al., 2020). Participants responded to items on a 5-point Likert-type scale from 1 (*disagree strongly*) to 5 (*agree strongly*). Three perfectionism factors are assessed by the BTPS-SF: rigid perfectionism (e.g., “*My opinion of myself is tied to being perfect*”), self-critical perfectionism (e.g., “*I have doubts about everything I do*”), and narcissistic perfectionism (e.g., “*I know I am perfect*”). Scores on each individual subscale were calculated through obtaining the mean of scores.

#### Self-Esteem

The 10-item Rosenberg Self-Esteem Scale (RSES; Rosenberg, 1965) is a 4-point Likert-type scale, from 1 (*strongly agree*) to 4 (*strongly disagree*). An example item includes “*I certainly feel useless at time*s.” The total score for self-esteem was computed by obtaining the sum of all 10 items (including five reverse-scored items), such that a higher score on the scale represented higher self-esteem.

### Study 3 Results

Descriptive statistics, Cronbach’s alpha reliability, and bivariate correlations for the total IPA score, three IPA subscales, and personality variables and self-esteem are presented in Table 9. Internal consistency reliabilities for the IPA Total and its three subscales were excellent (see Table 9). Correlations between impostor phenomenon and personality traits were also consistent with previous literature (Bernard et al., 2002; Casselman, 1991; Chae et al., 1995; Lester & Moderski, 1995; Ross et al., 2001). Imposter phenomenon was significantly positively associated with neuroticism and perfectionism (rigid, self-critical, and narcissistic), and significantly negatively associated with extraversion, agreeableness, and conscientiousness. Finally, IPA was significantly negatively associated with self-esteem, suggesting that those who reported lower self-esteem also reported higher impostor phenomenon.

**Table 9.** Descriptive Statistics and Bivariate Correlation Matrix.

Variable	*M*	*SD*	α	1	2	3	4	5	6	7	8	9	10	11	12	13
1. DA	4.15	0.84	.93	—												
2. PD	3.04	0.96	.93	**.65**	—											
3. SHB	3.63	0.87	.82	**.52**	**.59**	—										
4. Total IPA	3.67	0.76	.96	**.91**	**.90**	**.71**	—									
5. Extraversion	2.97	1.12	.69	−**.21**	−**.19**	−**.18**	−**.23**	—								
6. Agreeableness	3.50	0.91	.32	−**.14**	−**.16**	−**.19**	−**.18**	.07	—							
7. Conscientiousness	3.51	0.89	.46	−.06	−**.30**	−**.47**	−**.25**	.07	.06	—						
8. Neuroticism	3.68	1.07	.62	**.45**	**.22**	**.29**	**.39**	−**.21**	−.10*	.03	—					
9. Openness	3.50	0.93	.24	.00	.01	.08	.02	.01	.02	.04	.05	—				
10. Rigid Perfectionism	3.12	0.99	.83	**.36**	**.22**	**.13**	**.31**	−.02	−**.14**	**.16**	**.21**	−.03	—			
11. Self-critical Perfectionism	3.48	0.82	.83	**.70**	**.49**	**.42**	.**66**	−**.18**	−**.14**	−.04	**.46**	−.02	**.55**	—		
12. Narcissistic Perfectionism	2.11	0.72	.73	.03	**.19**	.08*	.**12**	.05	−**.35**	−.05	−.00	−.06	**.46**	**.17**	—	
13. Self-esteem	23.40	5.47	.88	−**.64**	−**.64**	−**.58**	−**.72**	**.28**	.**16**	.**25**	−**.39**	.05	−**.23**	−**.60**	−.01	—

*Note.* Bolded values significant at *p* < .01. IPA = Impostor Phenomenon Assessment; DA = Doubts About Achievement; PD = Perceived Discrepancy; SHB = Self-Handicapping Behaviors.

* Significant at *p* < .05.

#### Confirmatory Factor Analysis

To assess the factor structure of the IPA, we conducted confirmatory factor analysis and structural equation modeling using Mplus version 8 (Muthén & Muthén, 1998-2012). The fit statistics supported a three-factor model, χ2(699). = 3,348, *p<.*001. Findings suggested adequate fit per the RMSEA = 0.08 (90% CI = [.079, .085], *p <* .001) and the SRMR = 0.09 (Hu & Bentler, 1999; MacCallum et al., 1996). In addition, all loadings were significant at the *p < .*01 level. Although some fit statistics suggested adequate fit (TLI = 0.73, CFI = 0.75), it is possible that this is due to the parsimonious model favoring RMSEA and SRMR.

#### Exploratory Analyses

To assess for differences in gender, we conducted an independent samples t-test. Similar to Study 2, results for gender suggested significant differences between men and women on overall IPA and Doubts about Achievement; however, contrasting Study 2, no significant differences were found for Perceived Discrepancy and Self-Handicapping Behaviors (Table 10). To assess for differences across ethnicity and age, we conducted one-way ANOVAs. Contrasting Study 2, there were no significant differences in impostor phenomenon across ethnicity, *F*(5, 552) = 1.80, *p* = .11. Similarly, there were no significant differences across age, *F*(26, 477) = 1.26, *p =* .18.

**Table 10:** Differences Between Genders in Study 3.

Variable	Men (*n =* 151) *M* (*SD*)	Women (*n =* 387) *M* (*SD*)	*t*	*p*
DA	3.74 (0.90)	4.30 (0.76)	−7.20	.00**
PD	2.88 (0.93)	3.06 (0.95)	−1.95	.05
SHB	3.68 (1.03)	3.65 (1.09)	.33	.74
Total IPA	3.42 (0.82)	3.75 (0.76)	−4.53	.00**

*Note.* IPA = Impostor Phenomenon Assessment; DA = Doubts About Achievement; PD = Perceived Discrepancy; SHB = Self-Handicapping Behaviors.

** *p* < .01.

To explore the relation between objective academic success (GPA) and impostor phenomenon, we conducted a Pearson correlation analysis. Findings suggested that there were no significant relations between GPA and IPA (*r =* −.04, *p* = .35), DA (*r =* .02, *p =* .69), or PD (*r =* −.06, *p =* .17). However, findings did suggest a significant negative relation between GPA and SHB, suggesting that those who reported engaging in more self-handicapping behaviors also reported lower GPA (*r =* −.14, *p < .*01).

## General Discussion

Despite growing attention regarding the experience of feeling like a “fraud,” impostor phenomenon has received little empirical attention in terms of psychometric assessment and the dimensionality of its factors. With limited psychometric validation and factor structure present within existing measures of impostor phenomenon (Mak et al., 2019), concerns have also been raised about the conceptualization of this phenomenon. As such, the goal of the present research was to first provide clarification regarding the conceptualization of impostor phenomenon, and then establish a comprehensive, multidimensional measure to assess the described factor structure and foundational characteristics of impostor phenomenon.

To do so, we presented a theoretical framework through integrating themes present in the existing research literature. This led to conceptualizing impostor phenomenon as *the subjective experience of perceived self-doubt in one’s abilities and accomplishments compared with others, despite evidence to suggest the contrary*. In line with this conceptualization is a transcendent theme of self-doubt and comparison with others as motivating thoughts, feelings, and actions. This conceptualization, as well as the theoretical framework of impostor phenomenon, further guided subsequent item and scale development. The proposed factor structure of the new scale that emerged in Study 1 was replicated through confirmatory factor analyses in Studies 2 and 3 and supported three first-order factors (Doubts about Achievement, Perceived Discrepancy, and Self-Handicapping Behaviors).

The current findings support the notion that impostor phenomenon is a related, but distinct, phenomenon when considering in the context of personality, perfectionism, and self-esteem. Convergent validity was consistent with previous studies to suggest that the novel IPA is positively related to neuroticism, perfectionism, and low self-esteem, which have previously been identified as “maladaptive” traits (Bernard et al., 2002; Casselman, 1991; Chae et al., 1995; Cokley et al., 2018; Lester & Moderski, 1995; Ross & Krukowski, 2003; Ross et al., 2001). Mirroring previous research (e.g., Bernard et al., 2002; Chae et al., 1995; Ross et al., 2001), we found that impostor phenomenon was negatively related to extraversion, agreeableness, and conscientiousness, and not significantly related to openness. Together, these findings support the initial convergent and divergent validity of the novel IPA measure.

There is significant benefit to the development of this clarified conceptualization and assessment of impostor phenomenon. First, having a cohesive and multidimensional scale will allow for an increased understanding of the multifaceted nature of impostor phenomenon in a psychometrically valid format. In doing so, we will be able to identify impostor phenomenon more accurately and in a dimensional way, thus capturing the diverse presentations of this phenomenon. Second, increased identification and understanding of this phenomenon will allow for improved clinical identification, thus allowing for greater targeting within clinical treatment planning and personal goal setting. With particular attention to the nature of the various subdomains, this is promising in terms of theorizing successful treatment approaches, an area that has received little to no attention in the existing impostor phenomenon literature (Bravata et al., 2019). For example, “Doubts About Achievement” may be ideal for cognitive and emotion-focused intervention, the “Perceived Discrepancy” may be well suited for cognitive restructuring, and the “Self-Handicapping Behaviours” might be more effectively addressed through behavioral interventions including behavioral activation.

## Limitations

The current scale does not seek to differentiate those experiencing the subjective experience of *feeling* like a “fraud” from those who may be “real” impostors. To clarify, our conceptualization operationalizes impostor phenomenon as the subjective perception of not being competent *despite significant evidence to suggest otherwise*. This excludes those who are objectively, and intentionally, faking their accomplishments. “Real” impostors are those who intentionally present a false self with the goal of deliberately deceiving others (Kets de Vries, 2005; McElwee & Yurak, 2007). For example, a “real” imposter would be someone who lies on their resume about their educational qualifications with the intention of obtaining a specific occupational role for which they are not actually qualified. These “real” imposters may still experience fears of being exposed for their intentional misrepresentation of the self (Kets de Vries, 2005); however, this fear is objective in nature compared with the subjective and perceived incompetence of those experiencing impostor phenomenon who are otherwise objectively qualified. It is possible that having further information regarding objective achievement may allow further contrast to individuals’ attribution, beliefs about the self, and behaviors when experiencing either impostor phenomenon or “real impostorism.”

Given the assessment at a single timepoint, it is currently not possible to track the test–retest reliability nor stability of impostor phenomenon across time and even situations. Given the possible implications of the current scale for clinical intervention, it would be beneficial to develop a method of experientially tracking impostor phenomenon over time (i.e., over the course of a given day, throughout the school year, and across transitional periods). This would also aid in informing the developmental trajectory, stability, and predictive utility of impostor phenomenon, and provide further information about the state or trait nature of this construct. At present, no known research has examined impostor phenomenon longitudinally.

The current sample represents a limited demographic population, as well as an imposter-prone sample in an academic setting. Although there is minimal cross-sectional research suggesting mixed findings surrounding age effects (Bravata et al., 2019), there is some evidence to suggest that periods of transition typically represent increased stress and subjective difficulties adapting to new expectations and demands (Keefer, 2015). Recognizing the current samples of primarily younger aged, first year undergraduate students, the generalizability of this sample to the larger population is limited. Similarly, with the first-year undergraduate population (i.e., Study 1), it is likely that reported GPA at baseline was not an accurate representation of individual achievement (particularly given that many first-year undergraduate students had not yet received grades when baseline data were collected, and thus reported their high school GPA at the beginning of their university degree). There is some debate surrounding the extent to which high school GPA is related to university GPA (e.g., Elias & Macdonald, 2007). Previous research suggests that external factors including gender, stress, and self-esteem can differentiate academic success during the transition from high school to university (Wintre et al., 2011). Recognizing alternative ways of measuring success across academic programs (and particularly when considering undergraduate vs. graduate studies), it would be beneficial to compare individuals based on additional objective measures (i.e., performance review and observer/external rating) to gain further insight into the impact and prevalence of impostor phenomenon across different levels of objective achievement. Similarly, future research should seek to assess the validity of the current scale across time in external achievement-related settings (e.g., employees in a workplace), to replicate our current results and establish further validity outside of the academic setting.

Finally, our results support previous research regarding suggesting that students identifying as women (Brauer & Proyer, 2019; Cokley et al., 2015), and Asian students report the highest levels of impostor phenomenon (Cokley et al., 2013, 2017), particularly with differences in perceived doubts about achievement. Our results also suggest possible differences for other ethnicities, primarily those identifying as Indigenous. These findings support recent criticism of impostor phenomenon suggesting that societal pressures and expectations relating to success disproportionately impact women and minority populations (Tulshyan & Burey, 2021). As such, future research should further examine the experience of impostor phenomenon across a more diverse sample of genders and cultures to further elucidate these differences.

## Conclusion

The present research represents an important step forward in the understanding of impostor phenomenon and its assessment. Within the current studies, we sought to further the understanding of impostor phenomenon through developing a clear conceptualization and theoretical framework, and then developing a new measurement of the proposed factors and subdomains associated with the experience of feeling like an impostor—The IPA. The current progress toward conceptual clarity, factor structure, and initial psychometric validation of the novel IPA will hopefully support continued study and understanding of impostor phenomenon.

## Appendix A

### Impostor Phenomenon Assessment

Please read each of the following statements carefully and indicate how characteristic it is of your own experiences.

**Table:**

1 Strongly Disagree	2 Moderately Disagree	3 Somewhat Disagree	4 Somewhat Agree	5 Moderately Agree	6 Strongly Agree
**Doubts About Achievement**					
1. Even in situations for which I am well-prepared (e.g., studied very hard and long for an examination or worked tirelessly on a project), I still have doubts about my ability to perform well.					
2. I often feel that I have to work harder than others to achieve all that I do.					
3. I often get “down on myself” when I perform less than perfectly on a task or a problem.					
4. I often worry about not succeeding on a task, even though others around me have considerable confidence that I will do well.					
5. I often find myself putting more effort into tasks compared with others.					
6. When it comes to achieving and attaining goals, I suppose one might call me a “perfectionist”.					
7. Others have told me that I often do more than necessary when it comes to completing a task.					
8. I often compare my ability to those around me and believe they are more intelligent than I am.					
9. When people praise me for something I’ve accomplished, I’m afraid I won’t be able to live up to their expectations of me in the future.					
10. When I’ve succeeded at something and received recognition for my accomplishments, I have doubts that I can keep repeating that success.					
11. I often feel like I have to put more effort into my tasks because I am not as smart as those around me.					
12. I’m often afraid that I will fail at a new assignment or undertaking even though I generally do well at what I attempt.					
13. I often find myself spending more time than necessary in completing tasks or assignments.					
14. When I’m praised for something, I sometimes wonder if I will be able to do as well the next time.					
15. I feel discouraged if I’m not “the best” or at least “very special” in situations that involve achievement.					
16. I often worry about whether others will view me as a success or a failure.					
17. I tend to remember the incidents where I have not done my best more than those times that I have done my best.					
18. I often foresee failure when entering new situations that require a demonstration of my abilities.					
19. I avoid evaluations if possible and have a dread of others evaluating me.					
20. I try not to get too involved in competitive environments, so it won’t hurt so much if I lose or do poorly.					
21. I am often surprised when I perform well on a project or a test.					
22. When I receive a compliment, I find it difficult to accept the compliment, and often explain it away or give credit to others.					
23. When I receive a compliment about my academic or professional abilities, I sometimes find myself making excuses for explaining away the compliment.					
24. I’m afraid people important to me may find out that I’m not as capable as they think I am.					
25. Even though I feel that I have a lot of potential, I sometimes feel like an intellectual “fraud” or “phony.”					
26. If I’m going to receive a promotion or gain recognition of some kind, I hesitate to tell others until it is an accomplished fact.					
27. I often achieve success on a project or test when I have anticipated that I would fail.					
**Perceived Discrepancy**					
28. I feel that I have attained my present academic or professional position through “pulling strings” or “having connections.”					
29. I sometimes think I obtained my present position or gained my present success because I happened to be in the right place at the right time or knew the right people.					
30. I often feel I receive praise or grades that I don’t deserve.					
31. If I get a high grade on a work assignment, I tend to feel that I’ve fooled my teacher or supervisor.					
32. I obtained my present position because of something about me that I didn’t work for (e.g., coming from an underrepresented group).					
33. Sometimes, I believe that my success in my life or in my job has been the result of some kind of error.					
34. At times, I have felt I am in my present position or academic program through some kind of mistake.					
35. I often feel that my success has been due to some kind of luck.					
36. My achievements have been due more to external factors, such as luck or effort, rather than to my own inherent abilities.					
37. On some occasions when someone has praised me for something, I tend to feel that I fooled them.					
38. In some situations, I feel like a “great pretender”: that is, I’m not as genuine as others think I am.					
39. Sometimes, I am afraid I will be discovered for who I really am.					
40. I obtained my present position solely because of an affirmative action policy.					
41. I often feel I am concealing secrets about my abilities from others.					
42. I sometimes feel there’s something false or misleading about me that others don’t notice.					
43. I feel that there is a significant disparity between the “intellectual self” that others perceive and the “intellectual self” that I really am.					
44. I have sometimes convinced an important person that I am brighter or more talented than I really am.					
45. People tend to believe I am more competent than I really am.					
46. At a social event, I sometimes feel that I try to impress people by acting more intelligently than I really feel I am.					
47. Significant people in my life tend to believe that I am more academically or professionally competent than I really am.					
**Self-Handicapping Behaviors**					
48. I find myself often leaving tasks to the last minute.					
49. In preparing for deadlines, I often waste time doing other things.					
50. I typically delay getting started on tasks because I worry that I’m not up to the challenge.					
51. I worry about my ability to complete a task, and often end up delaying making decisions about the task until it is too late.					
52. Mostly, I find that I measure up to the standards that I set for myself. (r)					
53. My public and private self are the same person. (r)					
54. I rarely do a project or task as well as I’d like to do it.					

## References

[bibr1-10731911221141870] Ahlfeld A. J. (2009). The impostor phenomenon revisited: The intersection of race, gender, and professional status for women of color [Ph.D., Alliant International University San Diego].

[bibr2-10731911221141870] Austin C. C. Clark E. M. Ross M. J. Taylor M. J . (2009). Impostorism as a mediator between survivor guilt and depression in a sample of African American college students. College Student Journal, 43(4), 1094–1109.

[bibr3-10731911221141870] Badawy R. L. Gazdag B. A. Bentley J. R. Brouer R. L. (2018). Are all impostors created equal? Exploring gender differences in the impostor phenomenon-performance link. Personality and Individual Differences, 131, 156–163. 10.1016/j.paid.2018.04.044

[bibr4-10731911221141870] Bernard N. S. Dollinger S. J. Ramaniah N. V. (2002). Applying the big five factor personality factors to the impostor phenomenon. Journal of Personality, 78(2), 221–233. 10.1207/S15327752JPA7802_07 12067196

[bibr5-10731911221141870] Brauer K. Proyer R. T. (2019). The ridiculed impostor: Testing the associations between dispositions toward ridicule and being laughed at and the impostor phenomenon. Current Psychology. 10.1007/s12144-019-00262-5 33550593

[bibr6-10731911221141870] Brauer K. Wolf A. (2016). Validation of the German-language Clance Impostor Phenomenon Scale (GCIPS). Personality and Individual Differences, 102, 153–158. 10.1016/J.PAID.2016.06.071

[bibr7-10731911221141870] Bravata D. M. Watts S. A. Keefer A. L. Madhusudhan D. K. Taylor K. T. Clark D. M. Nelson R. S. Cokley K. O. Hagg H. K. (2019). Prevalence, predictors, and treatment of impostor syndrome: A systematic review. Journal of General Internal Medicine, 35(4), 1252–1275. 10.1007/s11606-019-05364-1 31848865 PMC7174434

[bibr8-10731911221141870] Bussotti C. (1990). The impostor phenomenon: Family roles and environment [Ph.D., Georgia State University].

[bibr9-10731911221141870] Casselman S. E. (1991). The impostor phenomenon in medical students: Personality correlates and developmental issues. Virginia Consortium for Professional Psychology.

[bibr10-10731911221141870] Chae J.-H. Piedmont R. L. Estadt B. K. Wicks R. J. (1995). Personological evaluation of Clance’s imposter phenomenon scale in a Korean sample. Journal of Personality Assessment, 65(3), 468–485. 10.1207/s15327752jpa6503_7 16367710

[bibr11-10731911221141870] Cheung L . (2018). Understanding imposter phenomenon in graduate students using achievement goal theory. [Ph.D. Fordham University]. https://research.library.fordham.edu/dissertations/AAI10824833

[bibr12-10731911221141870] Chrisman S. M. Pieper W. A. Clance P. R. Holland C. L. Glickauf-Hughes C. (1995). Validation of the Clance impostor phenomenon scale. Journal of Personality Assessment, 65, 456–467. 10.1207/s15327752jpa6503_6 16367709

[bibr13-10731911221141870] Clance P. Imes S . (1978). The imposter phenomenon in high achieving women: Dynamics and therapeutic intervention. Psychotherapy, 15, 241–247. 10.1037/H0086006

[bibr14-10731911221141870] Clance P. R. (1985). The impostor phenomenon: When success makes you feel like a fake. Peachtree Publishers. 10.1037/t11274-000

[bibr15-10731911221141870] Clance P. R. Dingman D. Reviere S. L. Stober D. R. (1995). Impostor phenomenon in an interpersonal/social context: Origins and treatment. Women & Therapy, 16(4), 79–96. 10.1300/J015v16n04_07

[bibr16-10731911221141870] Clance P. R. O’Toole M. A. (1987). The imposter phenomenon: An internal barrier to empowerment and achievement. Women & Therapy, 6(3), 51–64. 10.1300/J015V06N03_05

[bibr17-10731911221141870] Cokley K. Awad G. Smith L. Jackson S. Awosogba O. Hurst A. Stone S. Blondeau L. Roberts D. (2015). The roles of gender stigma consciousness, impostor phenomenon and academic self-concept in the academic outcomes of women and men. Sex Roles, 73(9–10), 414–426. 10.1007/s11199-015-0516-7

[bibr18-10731911221141870] Cokley K. McClain S. Enciso A. Martinez M. (2013). An examination of the impact of minority status stress and impostor feelings on the mental health of diverse ethnic minority college students. Journal of Multicultural Counseling and Development, 41(2), 82–95. 10.1002/j.2161-1912.2013.00029.x

[bibr19-10731911221141870] Cokley K. Smith L. Bernard D. Hurst A. Jackson S. Stone S. Awosogba O. Saucer C. Bailey M. Roberts D. (2017). Impostor feelings as a moderator and mediator of the relationship between perceived discrimination and mental health among racial/ethnic minority college students. Journal of Counseling Psychology, 64(2), 141–154. 10.1037/cou0000198 28277731

[bibr20-10731911221141870] Cokley K. Stone S. Krueger N. Bailey M. Garba R. Hurst A. (2018). Self-esteem as a mediator of the link between perfectionism and the impostor phenomenon. Personality and Individual Differences, 135, 292–297. 10.1016/j.paid.2018.07.032

[bibr21-10731911221141870] Comrey A. L. Lee H. B . (1992). A first course in Factor Analysis (2nd ed.). Lawrence Erlbaum.

[bibr22-10731911221141870] Cowman S. E. Ferrari J . (2002). “Am I for real?” Predicting impostor tendencies from self-handicapping and affective components. Social Behavior and Personality, 30, 119–126. 10.2224/SBP.2002.30.2.119

[bibr23-10731911221141870] Cozzarelli C. Major B . (1990). Exploring the validity of the impostor phenomenon. Journal of Social and Clinical Psychology, 9(4), 401–417. 10.1521/jscp.1990.9.4.401

[bibr24-10731911221141870] Craddock S. Birnbaum M. Rodriguez K. Cobb C. Zeeh S . (2011). Doctoral students and the impostor phenomenon: Am I smart enough to be here? Journal of Student Affairs Research and Practice, 48(4), 429–442. 10.2202/1949-6605.63

[bibr25-10731911221141870] Cromwell B. Brown N. Sanchez-Huceles J. Adair F . (1990). The impostor phenomenon and personality characteristics of high school honor a students. Journal of Social Behavior and Personality, 5(6).

[bibr26-10731911221141870] Cusack C. E. Hughes J. L. Nuhu N. (2013). Connecting gender and mental health to imposter phenomenon feelings. Psi Chi Journal of Psychological Research, 18(2), 74–81. 10.24839/2164-8204.JN18.2.74

[bibr27-10731911221141870] Devellis R. F. (2017). Scale development: Theory and applications (4th ed.). SAGE.

[bibr28-10731911221141870] Edwards P. W. Zeichner A. Lawler N. Kowalski R . (1987). A validation study of the Harvey Impostor Phenomenon Scale. Psychotherapy, 24, 256–259. 10.1037/H0085712

[bibr29-10731911221141870] Elias S. M. Macdonald S. (2007). Using past performance, proxy efficacy, and academic self-efficacy to predict college performance. Journal of Applied Social Psychology, 37, 2518–2531. 10.1111/j.1559-1816.2007.00268.x

[bibr30-10731911221141870] Ewing K. M. Richardson T. Q. James-Myers L. Russell R. K. (1996). The relationship between racial identity attitudes, worldview, and African American graduate students’ experience of imposter phenomenon. Journal of Black Psychology, 22(1), 53–66. 10.1177/00957984960221005

[bibr31-10731911221141870] Feenstra S. Begeny C. T. Ryan M. K. Rink F. A. Stoker J. I. Jordan J . (2020). Contextualizing the impostor “syndrome.” Frontiers in Psychology, 11, 575024. 10.3389/fpsyg.2020.575024 33312149 PMC7703426

[bibr32-10731911221141870] Feher A. Smith M. M. Saklofske D. H. Plouffe R. A. Wilson C. A. Sherry S. B. (2020). The Big Three Perfectionism Scale–Short Form (BTPS-SF): Development of a brief self-report measure of multidimensional perfectionism. Journal of Psychoeducational Assessment, 38(1), 37–52. 10.1177/0734282919878553

[bibr33-10731911221141870] Ferrari J. R. Thompson T. (2006). Impostor fears: Links with self-presentational concerns and self-handicapping behaviours. Personality and Individual Differences, 40(2), 341–352. 10.1016/j.paid.2005.07.012

[bibr34-10731911221141870] French B. F. Ullrich-French S. C. Follman D. (2008). The psychometric properties of the Clance Impostor Scale. Personality and Individual Differences, 44(5), 1270–1278. 10.1016/j.paid.2007.11.023

[bibr35-10731911221141870] Fried-Buchalter S. (1992). Fear of success, fear of failure, and the imposter phenomenon: A factor analytic approach to convergent and discriminant validity. Journal of Personality Assessment, 58(2), 368–379. 10.1207/s15327752jpa5802_13 16370869

[bibr36-10731911221141870] Fried-Buchalter S. (1997). Fear of success, fear of failure, and the imposter phenomenon among male and female marketing managers. Sex Roles, 37(11), 847–859. 10.1007/BF02936343

[bibr37-10731911221141870] Fujie R. (2010). Development of the state impostor phenomenon scale. Japanese Psychological Research, 52(1), 1–11. 10.1111/j.1468-5884.2009.00417.x

[bibr38-10731911221141870] Gravois J. (2007). You’re not fooling anyone. The Chronicle of Higher Education, 54(11), A1.

[bibr39-10731911221141870] Harvey J. C. (1981). The impostor phenomenon and achievement: A failure to internalize success [Ph.D., Temple University]. http://search.proquest.com/psycinfo/docview/303035505/abstract/A6B7B1398BC34A4DPQ/1

[bibr40-10731911221141870] Harvey J. C. Katz C. (1985). If I’m so successful why do I feel like a fake?. St. Martin’s Press.

[bibr41-10731911221141870] Hellman C. M. Caselman T. D. (2004). A psychometric evaluation of the Harvey Imposter Phenomenon scale. Journal of Personality Assessment, 83(2), 161–166. 10.1207/s15327752jpa8302_10 15456652

[bibr42-10731911221141870] Henning K. Ey S. Shaw D . (1998). Perfectionism, the impostor phenomenon and psychological adjustment in medical, dental, nursing and pharmacy students. Medical Education, 32. 10.1046/j.1365-2923.1998.00234.x 10211285

[bibr43-10731911221141870] Holmes S. W. Kertay L. Adamson L. B. Holland C. L. Clance P. R. (1993). Measuring the impostor phenomenon: A comparison of Clance’s IP scale and Harvey’s I-P scale. Journal of Personality Assessment, 60(1), 48–59. 10.1207/s15327752jpa6001_3 8433268

[bibr44-10731911221141870] Hu L. Bentler P. M. (1999). Cutoff criteria for fit indexes in covariance structure analysis: Conventional criteria versus new alternatives. Structural Equation Modeling: A Multidisciplinary Journal, 6(1), 1–55. 10.1080/10705519909540118

[bibr45-10731911221141870] Hutchins H. M. (2015). Outing the imposter: A study exploring imposter phenomenon among higher education faculty. New Horizons in Adult Education and Human Resource Development, 27(2), 3–12. 10.1002/nha3.20098

[bibr46-10731911221141870] Hutchins H. M. Rainbolt H. (2017). What triggers imposter phenomenon among academic faculty? A critical incident study exploring antecedents, coping, and development opportunities. Human Resource Development International, 20(3), 194–214. 10.1080/13678868.2016.1248205

[bibr47-10731911221141870] IBM Corp. Released 2019. IBM SPSS Statistics for Windows, Version 26.0. IBM Corp.

[bibr48-10731911221141870] Kaiser H. F. (1974). An index of factorial simplicity. Psychometrika, 39(1), 31–36. 10.1007/BF02291575

[bibr49-10731911221141870] Keefer J. M. (2015). Experiencing doctoral liminality as a conceptual threshold and how supervisors can use it. Innovations in Education and Teaching International, 52(1), 17–28. 10.1080/14703297.2014.981839

[bibr50-10731911221141870] Kets de Vries M. F. R. (2005). The dangers of feeling like a fake. Harvard Business Review, 83(9), 108–116. 16171215

[bibr51-10731911221141870] King J. E. Cooley E. L. (1995). Achievement orientation and the impostor phenomenon among college students. Contemporary Educational Psychology, 20, 304–312. 10.1006/ceps.1995.1019

[bibr52-10731911221141870] Kolligian J. Jr. Sternberg R. J. (1991). Perceived fraudulence in young adults: Is there an “imposter syndrome”? Journal of Personality Assessment, 56(2), 308–326. 10.1207/s15327752jpa5602_10 2056424

[bibr53-10731911221141870] Kumar S. Jagacinski C. M . (2006). Imposters have goals too: The imposter phenomenon and its relationship to achievement goal theory. Personality and Individual Differences, 40, 147–157. 10.1016/J.PAID.2005.05.014

[bibr54-10731911221141870] Lane J. A . (2015). The imposter phenomenon among emerging adults transitioning into professional life: Developing a grounded theory. Adultspan Journal, 14(2), 114–128. 10.1002/adsp.12009

[bibr55-10731911221141870] Langford J. (1990). The need to look smart: The impostor phenomenon and motivations for learning [Ph.D., Georgia State University—College of Arts and Sciences]. http://search.proquest.com/psycinfo/docview/303861336/abstract/EBD2F0837ED04ABCPQ/1

[bibr56-10731911221141870] Langford J. Clance P. R. (1993). The impostor phenomenon: Recent research findings regarding dynamics, personality and family patterns and their implications for treatment. Psychotherapy, 30(3), 495–501. 10.1037/0033-3204.30.3.495

[bibr57-10731911221141870] Leary M. R. Patton K. M. Orlando A. E. Wagoner Funk W. (2000). The impostor phenomenon: Self-perceptions, reflected appraisals, and interpersonal strategies. Journal of Personality, 68(4), 725–756. 10.1111/1467-6494.00114 10934688

[bibr58-10731911221141870] Leonhardt M. Bechtoldt M. N. Rohrmann S . (2017). All impostors aren’t alike – Differentiating the impostor phenomenon. Frontiers in Psychology, 8, 1505. 10.3389/fpsyg.2017.01505 28936188 PMC5594221

[bibr59-10731911221141870] Lester D. Moderski T. (1995). The impostor phenomenon in adolescents. Psychological Reports, 76(2), 466–466. 10.2466/pr0.1995.76.2.466 7667458

[bibr60-10731911221141870] Lige Q. M. Peteet B. J. Brown C. M. (2017). Racial identity, self-esteem, and the impostor phenomenon among African American college students. Journal of Black Psychology, 43(4), 345–357. 10.1177/0095798416648787

[bibr61-10731911221141870] MacCallum R. C. Browne M. W. Sugawara H. M. (1996). Power analysis and determination of sample size for covariance structure modeling. Psychological Methods, 1(2), 130–149. 10.1037/1082-989X.1.2.130

[bibr62-10731911221141870] Mak K. K. L. Kleitman S. Abbott M. J. (2019). Impostor phenomenon measurement scales: A systematic review. Frontiers in Psychology, 10, 671. 10.3389/fpsyg.2019.00671 31024375 PMC6463809

[bibr63-10731911221141870] McElwee R. O. Yurak T. J. (2007). Feeling versus acting like an impostor: Real feelings of fraudulence or self-presentation? Individual Differences Research, 5(3), 201–220.

[bibr64-10731911221141870] Muthén L. K. Muthén B. O. (1998-2012). Mplus user’s guide (6th ed.). Muthén & Muthén.

[bibr65-10731911221141870] Patzak A. Kollmayer M. Schober B. (2017). Buffering impostor feelings with kindness: The mediating role of self-compassion between gender-role orientation and the impostor phenomenon. Frontiers in Psychology, 8, 1289. 10.3389/fpsyg.2017.01289 28798714 PMC5526963

[bibr66-10731911221141870] Peteet B. J. Brown C. M. Lige Q. M. Lanaway D. A. (2015). Impostorism is associated with greater psychological distress and lower self-esteem for African American students. Current Psychology, 34(1), 154–163. 10.1007/s12144-014-9248-z

[bibr67-10731911221141870] Preston C. C. Colman A. M. (2000). Optimal number of response categories in rating scales: Reliability, validity, discriminating power, and respondent preferences. Acta Psychologica, 104(1), 1–15. 10.1016/S0001-6918(99)00050-5 10769936

[bibr68-10731911221141870] Rammstedt B. John O. P. (2007). Measuring personality in one minute or less: A 10-item short version of the Big Five Inventory in English and German. Journal of Research in Personality, 41(1), 203–212. 10.1016/j.jrp.2006.02.001

[bibr69-10731911221141870] Rohrmann S. Bechtoldt M. N. Leonhardt M. (2016). Validation of the impostor phenomenon among managers. Frontiers in Psychology, 7, 821. 10.3389/fpsyg.2016.00821 27313554 PMC4890534

[bibr70-10731911221141870] Rosenberg M. (1965). Rosenberg self-esteem scale. Database record, APA PsycTests. 10.1037/t01038-000

[bibr71-10731911221141870] Ross S. R. Krukowski R. (2003). The imposter phenomenon and maladaptive personality: Type and trait characteristics. Personality and Individual Differences, 34, 477–484. 10.1016/S0191-8869(02)00067-3

[bibr72-10731911221141870] Ross S. R. Stewart J. Mugge M. Fultz B. (2001). The imposter phenomenon: Achievement dispositions and the five-factor model. Personality and Individual Differences, 31(8), 1347–1355. 10.1016/S0191-8869(00)00228-2

[bibr73-10731911221141870] Sakulku J. Alexander J . (2011). The imposter phenomenon. International Journal of Behavioural Science, 6(1), 75–97.

[bibr74-10731911221141870] Seritan A. L. Mehta M. M. (2016). Thorny Laurels: The impostor phenomenon in academic psychiatry. Academic Psychiatry, 40(3), 418–421. 10.1007/s40596-015-0392-z 26152516

[bibr75-10731911221141870] Sonnak C. Towell T. (2001). The impostor phenomenon in British university students: Relationships between self-esteem, mental health, parental rearing style and socioeconomic status. Personality and Individual Differences, 31(6), 863–874. 10.1016/S0191-8869(00)00184-7

[bibr76-10731911221141870] Stein J. Y. Levin Y. Aloni R. Solomon Z. (2019). Psychiatric distress among aging decorated and non-decorated veterans: The role of impostorism and loneliness. Aging & Mental Health, 24(4), 582–590. 10.1080/13607863.2019.1594164 30938176

[bibr77-10731911221141870] Strube M. J . (1986). An analysis of the self-handicapping scale. Basic and Applied Social Psychology, 7(3), 211–224. 10.1207/s15324834basp0703_4

[bibr78-10731911221141870] Thompson T. Davis H. Davidson J. (1998). Attributional and affective responses of impostors to academic success and failure outcomes. Personality and Individual Differences, 25(2), 381–396. 10.1016/S0191-8869(98)00065-8

[bibr79-10731911221141870] Thompson T. Foreman P. Martin F. (2000). Impostor fears and perfectionistic concern over mistakes. Personality and Individual Differences, 29(4), 629–647. 10.1016/S0191-8869(99)00218-4

[bibr80-10731911221141870] Topping M. E. (1983). The impostor phenomenon: A study of its construct and incidence in university faculty members [Ph.D., University of South Florida].

[bibr81-10731911221141870] Topping M. E. Kimmel E. B. (1985). The imposter phenomenon: Feeling phony. Academic Psychology Bulletin, 7(2), 213–226.

[bibr82-10731911221141870] Tulshyan R. Burey J.-A. (2021). Stop telling women they have imposter syndrome. Harvard Business Review. https://hbr.org/2021/02/stop-telling-women-they-have-imposter-syndrome

[bibr83-10731911221141870] Valletta R. G . (2016). Recent flatterning in the higher education wage premium: Polarization, skill downgrading, or both? [Working paper, National Bureau of Economic Research, DOI 10.3386/w22935]

[bibr84-10731911221141870] Vaughn A. R. Taasoobshirazi G. Johnson M. L . (2020). Impostor phenomenon and motivation: Women in higher education. Studies in Higher Education, 45(4), 780–795. 10.1080/03075079.2019.1568976

[bibr85-10731911221141870] Want J. Kleitman S . (2006). Imposter phenomenon and self-handicapping: Links with parenting styles and self-confidence✫. Personality and Individual Differences, 40, 961–971. 10.1016/J.PAID.2005.10.005

[bibr86-10731911221141870] Wintre M. G. Dilouya B. Pancer S. M. Pratt M. W. Birnie-Lefcovitch S. Polivy J. Adams G. (2011). Academic achievement in first-year University: Who maintains their high school average? Higher Education, 62, 467–481. 10.1007/s10734-010-9399-2

